# IEWNet: Multi-Scale Robust Watermarking Network Against Infrared Image Enhancement Attacks

**DOI:** 10.3390/jimaging11050171

**Published:** 2025-05-21

**Authors:** Yu Bai, Li Li, Shanqing Zhang, Jianfeng Lu, Ting Luo

**Affiliations:** 1 School of Computer Science and Technology, Hangzhou Dianzi University, Hangzhou 310018, China; baiyu@hdu.edu.cn (Y.B.); lili2008@hdu.edu.cn (L.L.); jflu@hdu.edu.cn (J.L.); 2 Shangyu Institute of Science and Engineering, Hangzhou Dianzi University, Shaoxing 312365, China; 3 College of Science and Technology, Ningbo University, Ningbo 315211, China; luoting@nbu.edu.cn

**Keywords:** infrared images, image enhancement, multi-scale, robust watermarking, noise layer, enhancement sub-network

## Abstract

Infrared (IR) images record the temperature radiation distribution of the object being captured. The hue and color difference in the image reflect the caloric and temperature difference, respectively. However, due to the thermal diffusion effect, the target information in IR images can be relatively large and the objects’ boundaries are blurred. Therefore, IR images may undergo some image enhancement operations prior to use in relevant application scenarios. Furthermore, Infrared Enhancement (IRE) algorithms have a negative impact on the watermarking information embedded into the IR image in most cases. In this paper, we propose a novel multi-scale robust watermarking model under IRE attack, called IEWNet. This model trains a preprocessing module for extracting image features based on the conventional Undecimated Dual Tree Complex Wavelet Transform (UDTCWT). Furthermore, we consider developing a noise layer with a focus on four deep learning and eight classical attacks, and all of these attacks are based on IRE algorithms. Moreover, we add a noise layer or an enhancement module between the encoder and decoder according to the application scenarios. The results of the imperceptibility experiments on six public datasets prove that the Peak Signal to Noise Ratio (PSNR) is usually higher than 40 dB. The robustness of the algorithms is also better than the existing state-of-the-art image watermarking algorithms used in the performance evaluation comparison.

## 1. Introduction

In nature, all objects are capable of radiating infrared (IR) light. Furthermore, an IR image records the thermal radiation properties of the object. Thermal radiation properties are not visible to the human visual system, but the object can be imaged at any time based on the released energy. Moreover, it can be applied in some application environments such as disease screening [1], biotracking [2], robotic systems [3], disaster detection [4], and military [5].

However, IR images only record the temperature information of the object, IR images contain a limited amount of information. Additionally, the object exchanges heat with the surrounding environment, so, IR images always have the disadvantage of blurred edges. Therefore, IR images need to undergo a pre-processing step before further use. Infrared Enhancement (IRE) is one of the common pre-processing methods for IR images. It facilitates the subsequent image processing tasks by highlighting the edge information of the IR image, enhancing the contrast of the image pixels or removing the noise interference of the IR image. Initially, IR images were treated as two-dimensional arrays consisting of temperature values, and then various mathematical operations were applied to directly process the infrared images for better visualization. Later, image processing algorithms increasingly used frequency domain transforms. The infrared image is also decomposed into high-frequency, medium-frequency, and low-frequency subbands. The mathematical operations applied to the frequency domain subbands result in better IRE performance. With the popularization of machine learning, neural network models have become a hot research topic for IRE algorithms. Neural networks can intelligently learn the connection between the original IR image and the enhanced IR image. Nowadays, more and more algorithms combine frequency domain transform with neural network to enhance IR images.

There are three main categories for IRE algorithms: spatial domain, frequency domain, and hybrid domain-based methods [6]. Histogram Equalization (HE) algorithm is a common spatial domain IRE algorithm. Paul et al. [7] proposed the IRE algorithm based on fuzzy disparity HE algorithm. They obtained fuzzy disparity histogram based on the contextual information of the pixels. Then, they cropped and equalized the histogram of the IR image based on the extreme values of the different regions’ histograms. Wan et al. [8] used the particle wandering optimization algorithm to compute the entropy value of the local pixel blocks. This entropy value was applied to optimize the histogram of the local image, which in turn enhanced local details and overall contrast of the IR image. To avoid the problem of IR over-enhancement, Paul et al. [9] used logarithmic and power formulas to calculate the histogram.

With the development of science and technology, machine learning-based IRE algorithms in the spatial domain have become a hot research topic. Bhattacharya et al. [10] proposed an IRE model based on concise Convolutional Neural Networks (CNN). They applied 3 × 3 and 5 × 5 convolutional kernels to extract image features and added the classical Rectified Linear Unit (ReLU) activation function to refine the structure of the model. Kuang et al. [11] presented a novel deep learning model based on Generative Adversarial Network (GAN) for IRE task. The GAN model consumes more time and resources during the training process, but it has improved the enhancement effect of IR images.

In the literature, there are plenty of frequency domain transforms-based methods for IRE and image processing tasks. These transforms usually decompose original images to extract different features and reconstruct these features back to the original image with low error. Qi et al. [12] removed most of the noise information and weak signals using Fast Fourier Transform (FFT) domain for IR images. Zhao et al. [13] enhanced the IR image at smoothing transform scales and finally synthesized the complete IRE image. The hybrid domain-based IRE algorithms combine the previous two categories of IRE algorithms in both spatial and frequency domains. Wu et al. [14] enhanced the global contrast and local contrast of the IR image based on the histograms of the high-frequency and low-frequency subbands, respectively. Ein-shoka et al. [15] combined Discrete Wavelet Transform (DWT) with HE algorithm, and then they processed the histograms with the high-pass adaptive function.

The IRE algorithm enhances the contrast, edges, contours, and other features of the IR image. This improves the utility value of IR images, but at the same time, it also attacks the watermarked images. Therefore, this paper introduces a novel IR image robust watermarking algorithm under infrared enhancement attack. The encoder and decoder of the proposed algorithm use multi-scale structure to improve the robustness and imperceptibility of the algorithm. The algorithm applied two noise networks based on traditional and machine learning IRE algorithms to improve the robustness against IRE attacks. This is of great help in infrared image processing in industrial, scientific, and military fields.

In this paper, we propose an image watermarking auto-encoder model against the IRE attack based on conventional UDTCWT [16]. The proposed model improves the correct rate of watermark extraction from watermarked IRE images by adding the noise layer or enhancement module. The encoder and decoder of the model adopt the multi-scale neural network structure, and an attention mechanism is added after each of their convolutional layers to improve the feature’s extraction capability. The watermarked IR image output from the encoder is fed into the trained noise layer or enhancement module to simulate the effect of IRE attack, where the noise layer and enhancement module are used to simulate traditional and machine learning IRE algorithms, respectively. Finally, the decoder extracts the previously embedded watermark information in the IRE image. The main contributions of this work are as follows:

- We employ a multi-scale neural network structure to embed image watermarks of different scales onto the original IR image of the corresponding scale. The proposed model is able to select the appropriate watermark embedding location among features at different scales.
- We add a noise layer between the encoder and decoder to cope with the traditional IRE attack. The noise layer is used to simulate the principle of eight traditional IRE attacks. It is applied as the alternative to the eight traditional IRE attacks in the flow of the algorithm. This makes the proposed algorithm more robust against traditional IRE attacks.
- For machine learning IRE attacks where the specific structure is known, we train an enhancement sub-network to improve robustness. The enhancement sub-network is able to replace the role of four machine learning-based IRE algorithms in the IEWNet. The structure of the enhancement module is referenced to four classical IRE neural networks.

The rest of the paper is structured as follows: Section 2 introduces the research background of image robust watermarking algorithms. Section 3 describes the research work that is closely related to the proposed algorithm. Section 4 presents in detail the structure of the proposed auto-encoder, loss function, and training process. The experimental results that prove the relevant performance of the proposed algorithm are shown in Section 5. Finally, Section 6 and Section 7 summarize the strengths and weaknesses of the proposed algorithm and provides an outlook for future research work.

## 2. Background

IRE plays a very important role in improving the quality of IR images. However, IR images often have a significant connection with the research results of a group or enterprise. In order to prevent undesirable behavior such as data leakage, groups or companies that are copyright owners often embed digital watermarks into their IR images. Some companies want to produce important work equipment at low cost. They steal infrared images of industrial equipment to imitate the equipment and claim that the infrared image belongs to them. Other organizations enhance infrared images to prevent copyright holders from proving the origin of the image and to confuse the ownership of the image. The processing of watermarked IR images by IRE affects the extraction of digital watermarks, which in turn affects the copyright protection of IR images. Therefore, the image watermarking algorithm that can resist IRE attacks is necessary. The watermarking algorithm seeks the ability to resist image attacks, so we should consider a robust watermarking algorithm.

Image robust watermarking algorithms can also be categorized into three categories: spatial domain, frequency domain, and hybrid domain-based algorithms [17]. In recent years, image watermarking algorithms based on machine learning in the spatial domain are more common. Wang et al. [18] proposed a Deep Neural Network (DNN) model which contains encoder, discriminator, detector, and decoder. Singh et al. [19] incorporated a denoising module in a CNN-based auto-encoder for image watermarking. Boujerfaoui et al. [20] proposed an end-to-end DNN for print-shooting attacks, called Cam-Unet.

Apart from neural networks, image watermarking algorithms in the spatial domain based on traditional methods are also available. Xiao et al. [21] proposed a watermarking algorithm for screen-shooting images based on the matrix operation for screen image watermarking algorithm. Hasan et al. [22] applied Pascal’s triangle algorithm to look for the watermark embedding location. For the frequency domain-based algorithm, Peng et al. [23] extracted features in the Discrete Non-separable Shearlet Transform (DNST) domain of the image using Pseudo-Zernike Moments (PZM). The statistical model is then applied to compute several parameters to represent the extracted features. Zeng at al. [24] proposed a medical image watermarking algorithm based on KAZE features with Discrete Cosine Transform (DCT). Furthermore, Kumar et al. [25] combined DCT with DWT and then embedded both image watermarks in the frequency domain. DWT can also be applied in conjunction with the Walsh Hadamard Transform (WHT) and Singular Value Decomposition (SVD), and this frequency domain-based algorithm can be used to achieve robust and imperceptible image watermarking algorithms in the Y-channel of YCbCr images [26]. Devi et al. [27] proposed the H-Grey adaptive image watermarking embedding algorithm based on DWT and SVD in both frequency domains. Su et al. [28] employed Graph Based Transform (GBT) to count the distribution of stable features in the image.

Further, recent hybrid domain-based robust watermarking algorithms are often made by combining traditional frequency domain transforms and CNN. Zhang et al. [29] proposed a hybrid domain image auto-encoder based on FFT with diffusion model. Gorbal et al. [30] proposed a high-capacity image watermarking algorithm by combining the Nondownsampled Contour Wave Transform (NCWT), DWT, and DNN.

## 3. Related Work

In this paper, the machine learning model based on multi-scale feature extraction and the enhancement module are the two main prominent innovations of the proposed algorithm. The multi-scale algorithm can extract important features of the image more comprehensively, whereas the enhancement module can help the decoder to extract watermarking information more accurately after the watermarked IR image is processed by the machine learning-based IRE algorithm. In this section, the existing multi-scale-based algorithms are introduced along with the existing CNN-based IRE models.

### 3.1. Multi-Scale-Based Methods

Multi-scale algorithms are the mature technique in the image processing region. The Gaussian pyramid downsamples the image sequentially from the largest resolution to obtain a series of progressively smaller scales. Olkkonen et al. [31] proposed Gaussian pyramid transforms which can be applied for both 2D and 3D images. This frequency domain transform not only decomposes the image to extract features, but also reconstructs the obtained features into the original image with relatively low error. Yan et al. [32] applied Gaussian pyramid to the visible and IR image fusion algorithm. The algorithm first decomposes the visible and IR images into two scales. The first scale contains the number of subbands equal to the level of image decomposition. The second scale uses the Gaussian blurring algorithm to obtain the detailed features and base features of the image. The algorithm filters and combines the high- and low-frequency information of two images and finally produces a high-quality fused image. In order to reconstruct the Gaussian pyramid low-scale image in the maximum extent of the high-scale original image, the researchers proposed the Laplace pyramid algorithm. Gaussian pyramid performs Gaussian blurring before downsampling, then the low-level scale image contains only low-frequency information. The Laplace pyramid helps to reconstruct the resolution of the degraded image into a high-resolution image by calculating the difference with the high-level scale image to obtain the high-frequency information. Lai et al. [33] proposed a CNN for super-resolution tasks based on Laplace pyramid. Each low-scale image of the Laplace pyramid is fed into the network to predict the corresponding high-scale image. Furthermore, the network applies the Charbonnier loss function with high accuracy to supervise the training of the network. The algorithm combines the traditional Laplace pyramid with novel machine learning algorithms, which makes the algorithm outperform several classical machine learning super-resolution models in terms of speed and capacity. Multi-scale algorithms are also used in machine learning-based robust image watermarking algorithms. Qin et al. [34] proposed an end-to-end machine learning model for print-shooting attacks. The algorithm builds a deep noise simulation network after the encoder to learn the process of the image being printed and photographed. The model’s encoder processes the input image into tensors of different sizes through a convolutional operation, and then the input image is downsampled into different degrees to concatenate these tensors. This encoder based on multi-scale algorithm enhances the quality of the watermarked image and implies that the imperceptibility of the first algorithm is improved. Moreover, Rai et al. [35] also introduced a multi-scale algorithm in the model’s decoder and the watermarking information output from each scale is supervised by the baseline truth values during model training. The tensors generated by the multi-scale algorithm in the encoder are also fused with each other through downsampling and upsampling operations. The downsampling operation serves to reduce the training cost, prevent overfitting, and increase the sensory range while generating multiple scales. The upsampling operation is able to preserve some of the detailed features of the previous scale. This two-way propagation multi-scale algorithm is able to refer to the features of other scales during the training process of each scale, which in turn improves the accuracy of model training. The proposed IEWNet model structure is based on the multi-scale machine learning model structure presented by Rai et al. [35].

### 3.2. Infrared Enhancement-Based Networks

In recent years, machine learning-based IRE algorithms have become more and more common. The IRE-based CNN model introduced by Choi et al. [36] is applied to refine the task of nighttime target recognition in the field of autonomous driving. The network structure consists of a total of four convolutional layers with two 3 × 3 convolutional layers, one 5 × 5 convolutional layer, and one 7 × 7 convolutional layer. A ReLU activation function is set between each two convolutional layers to mitigate the problems of gradient vanishing and overfitting. Furthermore, Lee et al. [37] proposed a CNN-based IRE model for target detection based on luminance domain and residual structure. The model is fed with low-quality luminance images, and the output is trained with a high-quality baseline true value image. The feature extraction module first extracts the initial features of the luminance image using two 3 × 3 convolutional layers. Then, the mapping module performs dimensionality reduction using 1 × 1 convolutional layers and enhances the reduced features with nonlinear mapping. After the extension module expands the dimensionality of the features, the image reconstruction module outputs a high-quality image based on the residual structure. Moreover, Kuang et al. [38] introduced an IRE-based DNN for removing noise interference in IR image. The network applies hopping connections several times to prevent the loss of image information, and consists of denoising network and conditional discriminator. The denoising network consists of eight convolutional layers and eight inverse convolutional layers where each convolutional kernel has a size of 4 × 4, whereas the conditional discriminator consists of five 4 × 4 convolutional layers. Unlike normal discriminators that only input true and false images, the conditional discriminator also adds noise images as input. Additionally, Zhong et al. [39] employed three branches for feature extraction module to extract features from IR images, where each branch contains one or two convolutional layers and a ReLU activation function. The features of the three branches are concatenated into a tensor which is then fed into the image enhancement module. The image enhancement module first processes the input tensor using eight 3 × 3 convolutional layers, and the results of each convolutional layer are finally combined together. After dimensionality reduction by a 5 × 5 convolutional layer, the current tensor is applied for residual computation with the feature extraction module. Finally, the image enhancement module outputs the IRE image with one convolutional layer. In this paper, the proposed enhancement module is constructed with special reference to the above-mentioned IRE-based networks in the process of introducing the noise layer of the model. This will improve the robustness of the proposed algorithm against IRE attacks. The models proposed by [37,38,39,40] are referred to as, in order, Thermal Image Enhancement using CNN (TEN), Brightness-Based CNN (BCNN), Optical Noise Removal Deep CNN (ONRDCNN), and Auto-Driving CNN (ACNN) in the experimental results of Section 4.

## 4. Materials and Methods

In order to improve the ability of infrared image robust watermarking algorithms to resist IRE attacks, this paper constructs a multi-scale self-encoder based on infrared images for both traditional and machine learning IRE attacks. The infrared image datasets for the algorithm include GTOT [40], Road-Scene [41], TNO [42], LasHeR [43], RGBT210 [44], and RGBT234 [45].

**Figure 1:**
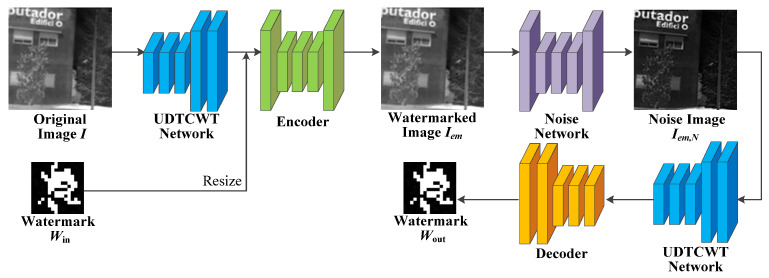
General network architecture of IEWNet.

The proposed IRE robust watermarking network (IEWNet) uses the encoder–decoder structure to embed and extract the watermarking information, respectively. Before the watermarked image is fed to the decoder, the noise layer or enhancement module simulates traditional or machine learning IRE algorithms to process the watermarked image. This section first introduces the network structure of the proposed IEWNet. Next, the details of training IEWNet are described in terms of loss function and training process. The general architecture of the proposed IEWNet is shown in Figure 1. The original infrared image I is first input to the UDTCWT [16] network U for preprocessing and is embedded with the watermark Win by the encoder E. Then, the noise network outputs the watermarked image Iem as the noisy image $I_{em,N}$. Finally, the UDTCWT [16] network U and the decoder D extract the watermarking information from the noisy image $I_{em,N}$.

### 4.1. Network Architecture

The proposed network consists of four sub-modules in total: UDTCWT [16] network U, encoder E, noise network N, and decoder D. The network takes the original IR image I as an input, and the UDTCWT [16] network U works with the encoder E to obtain the watermarked image $I_{em}$. After the noise network N outputs the noisy image $I_{em,N}$, the UDTCWT [16] network U works with the decoder D to obtain the extracted watermarking information $W_{\mathrm{out}}$.

**Figure 2:**
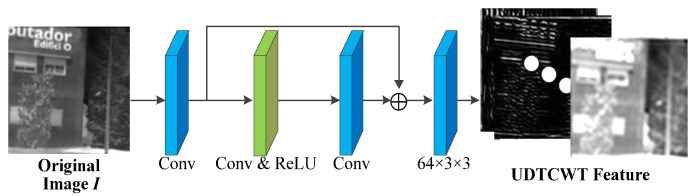
Network structure diagram for the UDTCWT [1] network *U*.

**Figure 3:**
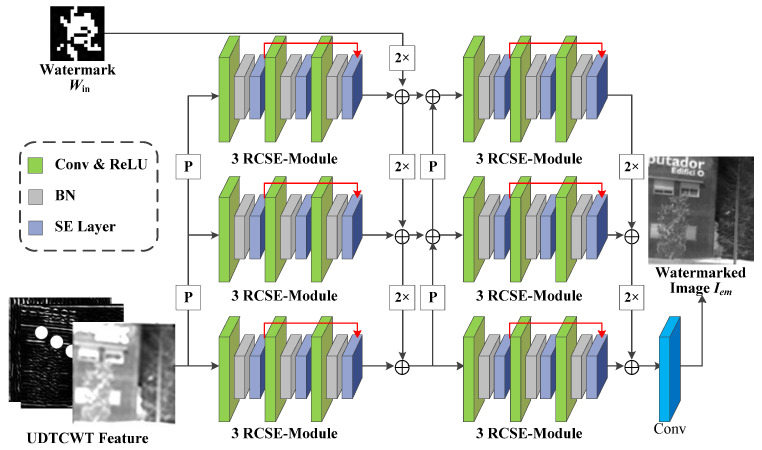
Network structure diagram for the encoder *E*.

**Figure 4:**
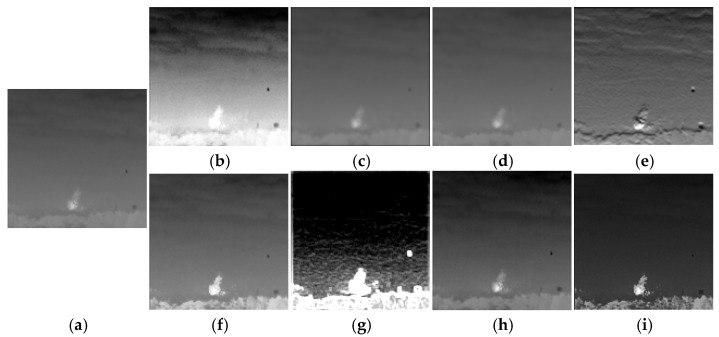
Example for eight traditional-based IRE attacks. (**a**) Orginal image. (**b**) HE. (**c**) Mean filter. (**d**) Median filter. (**e**) Sobel operator. (**f**) AHPBC. (**g**) DWT-EPSO. (**h**) DeepVIP. (**i**) ANFLC.

(1) UDTCWT [16] Network U: To make the frequency domain transform more suitable for incorporation into the proposed IEWNet, we train a small CNN with subbands of the UDTCWT [1] decomposition as targets. The main structure of UDTCWT [16] network U is shown in Figure 2. The network structure starts with the convolutional layer, followed by a residual structure consisting of a convolutional layer and a ReLU activation function. The final output from a convolutional layer is a 28-dimensional tensor. The convolutional kernel size is 3 × 3 and has 64 dimensions.

(2) Encoder E: Figure 3 shows the network structure for the encoder E, where “P” stands for 2 × 2 average pooling operation, “2×” stands for 64 × 3 × 3 doubly expanded convolutional layers, and (+) indicates the addition operation used to sum the features at each scale. Encoder E aims to encode the input UDTCWT [16] features with the original IR image $I_{co}$ into a fixed dimensional tensor and to extract multi-scale image features while reducing the dimensionality of the input. The structure of encoder E is generally divided into three scales. On the one hand, the UDTCWT [16] subnet extracts features after one or two 1/2 downsampling to form three dimensionality-decreasing features. On the other hand, a doubly extended convolutional layer can scale the watermarked image to twice or four times its size. Then, their sizes at these three scales will be in equal relationship. This will facilitate the combination of features at different scales with the help of downsampling and upsampling operations as illustrated in Figure 3. The inter-scale feature fusion will preserve more features from the original image I during the model training process, which will improve the imperceptibility of the encoder E. The Residual Convolutional SE (RCSE) module is a residual network structure consisting of three SE-Conv sub-blocks. The structure of the SE-Conv sub-blocks is based on channel attention module proposed by Cao et al. [46]. The ReLU activation function is capable of applying nonlinear operations on the model weights. The BN operation reduces the covariate bias of the model weights. After the tensor is processed sequentially by the 3 × 3 convolutional layer, ReLU activation function, and BN operation, the channel attention module is able to adjust the weights of the image watermark’s feature in different channels according to the important relationship between the channels. The attention module first reduces the dimension of the tensor to one dimension using the global average pooling algorithm. Then, two sets of Fully Connected (FC) layers with activation functions adjust the one-dimensional tensor. Finally, the output of the attention module is multiplied by the input tensor to achieve the final result. After the encoder E obtains the sum of the features at each scale, it outputs the watermarked IR image $I_{em}$ through a 64-dimensional 3 × 3 convolutional layer.

(3) Noisy Network N: In order to learn some patterns of IRE attacks, we set up the noise network N between the encoder and decoder to simulate these attack methods. IRE attacks are categorized into traditional-based methods and machine learning-based methods. For traditional-based methods, we choose eight attacks for IRE images as the target for model training. These attacks include HE, mean filter, median filter, and Sobel operator, and there are also four IRE attacks provided by the GitHub public repository: Adaptive Histogram Partition and Brightness Correction (AHPBC) [47], Discrete Wavelet Transform and Event triggered Particle Swarm Optimization (DWT-EPSO), DeepVIP [48], and Adaptive Non-local Filter and Local Contrast (ANFLC) [49]. The code for these algorithms is all using Matlab. Figure 4 illustrates the results of eight traditional IRE algorithms.

**Figure 5:**
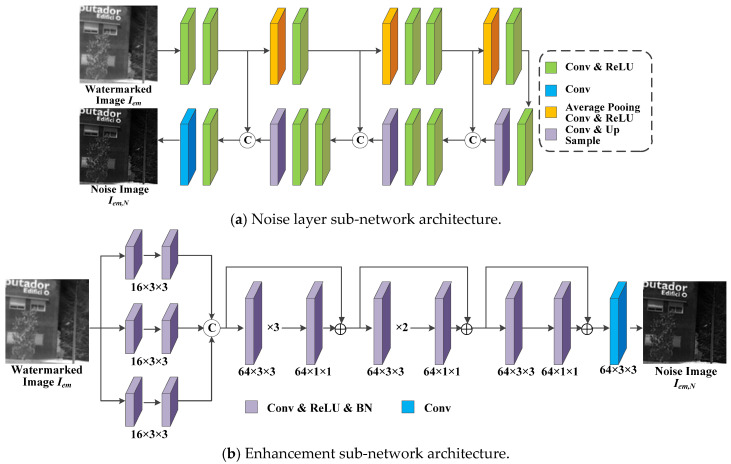
Network architecture diagram (**a**,**b**) for the proposed noise network *N*.

The noise layer in Figure 5a is applied to machine learn these eight conventional IRE algorithms. The noise layer refers to Unet [50] and uses 2 × 2 maximum pooling with the doubled expansion of the convolutional layer for upsampling and downsampling operations. Furthermore, it uses combination of convolutional layers and ReLU activation functions to continuously extract the desired features. Based on the basic idea of the four IRE-based models [36,37,38,39] introduced in the details of Section 3.2 in constructing the enhancement network, the proposed enhancement sub-network first extracts features from different receptive fields by three different sizes of convolutional kernels, where the convolutional kernels are 7 × 7, 5 × 5, and 3 × 3. The extracted features are sequentially processed by three residual structures, and each residual structure consists of four, three, and two convolutional blocks, respectively. Each convolutional block includes a convolutional layer, a ReLU activation function and a BN operation. The specific structure of the model is shown in Figure 5b.

**Figure 6:**
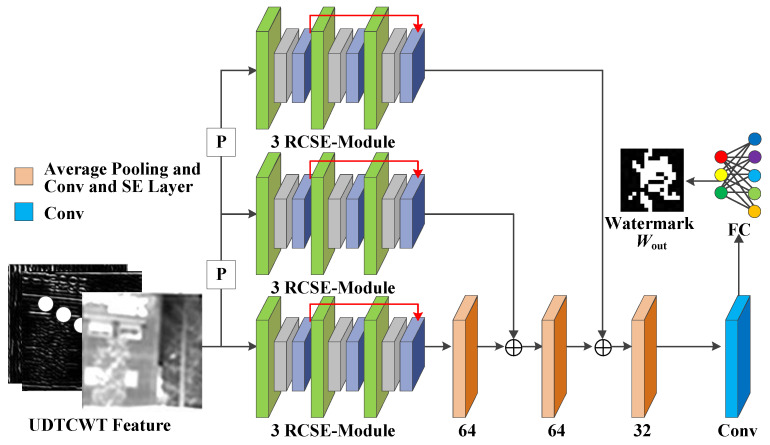
Network architecture for the decoder *D*.

(4) Decoder D: Figure 6 illustrates the structure of decoder D. Decoder D decodes the noisy image $I_{em,N}$ and maps the features flowing through the other sub-networks to the watermark information. Unlike the encoder E, the decoder D has only one residual convolution module per scale. Both the encoder E and the decoder D have exactly the same structure for their residual convolution modules. The first scale undergoes two 2 × 2 average pooling operations with SE-Conv blocks to match the features of the other two scales in turn. To obtain the mapping of the features to the watermark image $W_{\mathrm{out}}$, the decoder D applies a FC layer at the end. We choose the scale with the smallest size to output the watermark image $W_{\mathrm{out}}$, because the FC layer maps each weight of the model. If the number of weights in the feature tensor is too large, the difficulty of model training increases substantially.

### 4.2. Loss Function

All four subnets of IEWNet apply the L2 loss function, which is the Mean Squared Error (MSE) loss function. $\theta_{U}$, $\theta_{E}$, $\theta_{N}$, and $\theta_{D}$ denote the network weights of UDTCWT [1] network U, encoder E, noise network N, and decoder D, respectively. The UDTCWT [1] network U will model the decomposition process of the UDTCWT [1] transform. Furthermore, it is more suitable as a network model for joint training with other networks. The loss function $L_{U}$ for UDTCWT [1] network U is calculated as follows:

(1) $$L_{U}=\left\{\begin{array}{cc} \mathrm{MSE}\left(\mathrm{UDTCWT}\left(I\right),U\left(\theta_{U},I\right)\right), & \;\mathrm{if}\;\mathrm{Encoder}\\ \mathrm{MSE}\left(\mathrm{UDTCWT}\left(I_{em}\right),U\left(\theta_{U},I_{em}\right)\right), & \;\mathrm{if}\;\mathrm{Decoder} \end{array}\right..$$

The encoder E loss minimizes the error between the original IR image I and the watermarked IR image $I_{em}$. Equation (2) introduces loss function $L_{E}$ of the encoder E.

(2) $$L_{E}=\mathrm{MSE}\left(I,I_{em}\right)=\mathrm{MSE}\left(I,E\left(\theta_{E},U\left(\theta_{U},I\right)\right)\right).$$

In order to learn the principles of multiple IRE algorithms, the loss function $L_{N}$ of the noise network N will make the outputs of the noise layer and enhancement module be closer to these IRE algorithms. The structure of the noise layer and the enhancement module are different, however, they both use the same loss function as shown in Equation (3).

(3) $$L_{N}=\mathrm{MSE}\left(I_{E,co},I_{N}\right)=\mathrm{MSE}\left(I_{E,co},N\left(\theta_{N},I_{em}\right)\right).$$

In this case, $I_{E,co}$ is the IRE image produced by traditional and machine learning IRE algorithms based on the original image I.

The loss function $L_{D}$ of the decoder D continuously improves the similarity between the output watermark $W_{\mathrm{out}}$ and the input watermark $W_{\mathrm{in}}$ during the training process. The UDTCWT [1] network U also helps decoder D to extract more features of the watermarked IR image $I_{em}$. Equation (4) calculates the error between the input watermark $W_{\mathrm{in}}$ and the output watermark $W_{\mathrm{out}}$.

(4) $$L_{D}=\mathrm{MSE}\left(W_{\mathrm{in}},W_{\mathrm{out}}\right)=\mathrm{MSE}\left(W_{\mathrm{in}},D\left(\theta_{D},\theta_{U}\left(I_{em}\right)\right)\right).$$

Finally, the loss function L of the proposed IEWNet during joint training is

(5) $$L=L_{E}+\delta_{1}L_{D},$$

where $\delta_{1}$ is the hyperparameter that controls the percentage of the equilibrium loss function.

### 4.3. Training Process

The UDTCWT [16] network U and the noise network N of the proposed IEWNet work separately for network training. The encoder E and the decoder D are jointly trained together. For the network training, we use six public IR images datasets: GTOT [40], Road-Scene [41], TNO [42], LasHeR [43], RGBT210 [44], and RGBT234 [45]. These datasets include IR images of different times, locations and targets. The number of images in the training and testing sets is 80% and 20% of the whole dataset, respectively. The size of all IR images are scaled or randomly cropped to 128 × 128. Training UDTCWT [16] network U requires that the original IR image I is first decomposed into 28 subbands by using UDTCWT [16] transform. The loss function $L_{U}$ makes the output of the network closer and closer to these 28 subbands. The noise layer of the noise network N is trained based on the IRE image generated by the traditional-based IRE algorithms. On the other hand, the enhancement sub-network, is trained on the randomly selected dataset from references [36,37,38,39]. The loss function used in the training process for both networks of the noise network is $L_{N}$.

We jointly train the encoder E and decoder D while keeping the network weights of the UDTCWT [16] network U and the noise network N unchanged. The original IR image I is first preliminarily extracted with features by the UDTCWT [16] network U. The watermarked IR image $I_{em}$ is fed into the noise layer to simulate the process of traditional-based IRE algorithms. These features and the original image watermark $W_{\mathrm{in}}$ are used as carriers by encoder E to generate the watermarked IR image $I_{em}$. The watermarked IR image $I_{em}$ is fed into the noise layer to simulate the process of the eight traditional-based IRE algorithms, or it can be applied as an input carrier for the enhancement sub-network to simulate the result of the four machine learning-based IRE algorithms [36,37,38,39]. The output of the noise networks FN N results in the noisy image $I_{em,N}$. The UDTCWT [16] network U also extracts roughly the features of the noisy image $I_{em,N}$ and these features are used by the extraction of the final image watermark $W_{\mathrm{out}}$. The joint training applies the loss function L as a supervised metric for the training to obtain the encoder E and decoder D that the algorithm expects.

## 5. Experimental Results

This section first describes the dataset used, software and hardware needed for the proposed algorithm. Then, it lists the performance evaluation, imperceptibility experimental results and robustness of the UDTCWT [16] transform.

### 5.1. Datasets and Experimental Configuration

**Figure 7:**
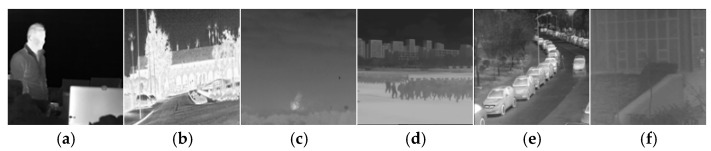
Sample IR images from the six datasets. (**a**) GTOT, (**b**) Road-Scene, (**c**) TNO, (**d**) LasHeR, (**e**) RGBT210, (**f**) RGBT234.

**Figure 8:**
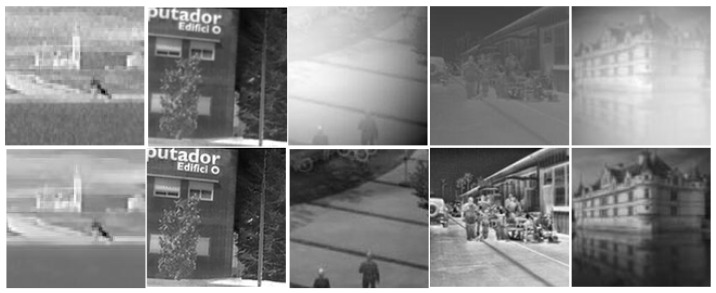
Training set of IR images for the augmented sub-network. The first row is the original IR image. The second row is the enhanced infrared image.

To verify the effectiveness and generalization of the proposed IEWNet, we select six public IR image datasets in Figure 7: GTOT [40], Road-Scene [41], TNO [42], LasHeR [43], RGBT210 [44] and RGBT234 [45]. These datasets include human infrared images in both indoor and outdoor environments. And the infrared images of outdoor environments are also available for buildings and vehicles in the city as well as in the field. These infrared images are taken during the day and night. They cover lots of situations in time and space. They form a total training set for model training. These datasets have both visible and IR images at the corresponding time. Since our model is proposed to overcome IRE attacks, we only consider IR images from these six datasets. First, the IR images are randomly filtered, cropped or scaled and then the image size is adjusted to 128 × 128. Figure 7 illustrates sample images from six datasets. In order to ensure good model training effect, we control the ratio of the number of infrared images between the training set and the test set at 4:1. and with reference to the number of images in various datasets, this paper screens different numbers of infrared images for each dataset. The training set of the six datasets has 80, 160, 80, 160, 160, 160, and 80 images, respectively. The number of images in the test set is set to be 1/4 of the training set. The watermarking information is an image consisting of a 256-bit with 0,1 sequence. In training the enhancement module of the noise network N, 100 IR images are randomly selected from the four algorithms presented in [36,37,38,39] as the training set. The settings of this training set are consistent with the six datasets. Figure 8 illustrates five IR images from the training set of the augmented sub-network. All experiments are conducted on a computer Dell OptiPlex 7070 with an Intel(R) Core(TM) i7-9700 CPU processor and with NVIDIA GeForce GTX 1660 graphics card from Randrock, Texas, USA. Furthermore, we use MATLAB R2016a and PyCharm 2020.3.3 to build our proposed network. In experiments, our model is trained on 100 epochs using Stochastic Gradient Descent (SGD) with a momentum of 0.9, a batch size of 1, and the initialization learning rate and weight decay rate are set to 0.001.

### 5.2. UDTCWT

**Figure 9:**
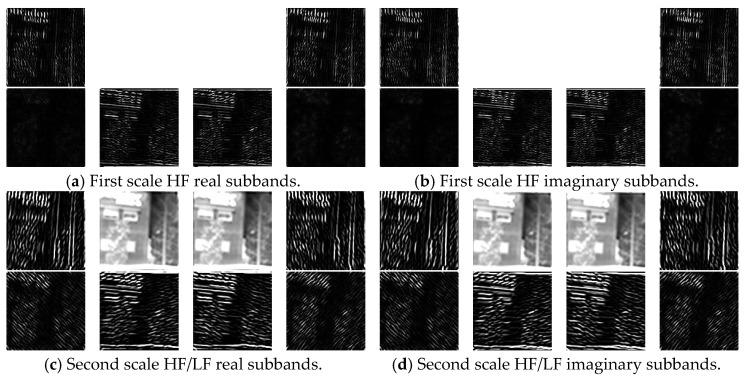
All subbands resulting (**a**–**d**) from the secondary UDTCWT [16] decomposition of an IR image.

The UDTCWT [1] extracts high-frequency subbands at different scales from the IR image in a total of six directions at ±15°, ±45°, and ±75°. In this paper, the application of the secondary UDTCWT [16] transform produces two scales. We take the second original IR image in Figure 9 to train UDTCWT [1] network U, and all the high-frequency (HF) and low-frequency (LF) subbands are shown in Figure 9.

### 5.3. Imperceptibility Comparison

**Figure 10:**
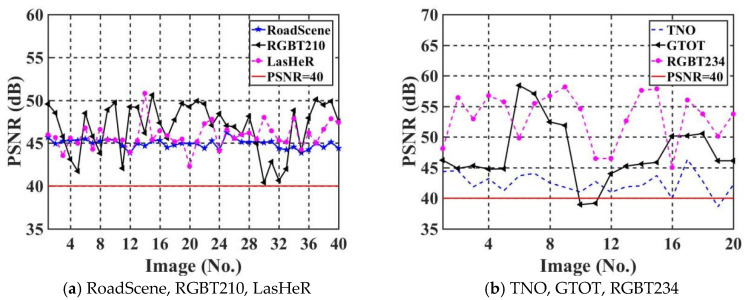
Comparison of PSNR values for the six test sets (**a**,**b**).

The imperceptibility of the proposed algorithm is evaluated by using PSNR. The PSNR value quantifies the degree of loss in image quality. The higher the PSNR value is, the higher the quality of the watermarked IR image $I_{em}$, and the watermarked IR image $I_{em}$ tended to be more similar to the original IR image I. Figure 10 shows PSNR values of the test set for six IR images datasets, where the horizontal and vertical coordinates of each graph represent the image serial number and the corresponding PSNR value of the image, respectively. We choose PSNR = 40 as the standard line for evaluating image quality.

(6) $$\mathrm{PSNR}=10\times \log_{10}\frac{\mathrm{length}\left(I\right)\times \mathrm{width}\left(I\right)\times 255\times 255}{\left[\sum_{i=1}^{\mathrm{length}\left(I\right)}\sum_{j=1}^{\mathrm{width}\left(I\right)}{\left(I_{em}-I\right)}^{2}\right]/\mathrm{Size}\left(I\right)},$$

where “length()” denotes the length of the image. “width()” denotes the width of the image. “Size()” is the area of the image. The experimental results demonstrated in Figure 10 prove that nearly all the PSNR values of the watermarked images are greater than 40. This proves that the proposed IEWNet network has a good imperceptibility.

### 5.4. Robustness Comparison

Imperceptibility is an indicator that evaluates the difference between the carrier after embedding the watermarked information and the original carrier as perceived by the human eye. If the difference between the two carriers is very small, most of the index values of imperceptibility will be larger. The smaller the difference between the watermarked infrared image $I_{em}$ and the original infrared image I is, the better the imperceptibility of the watermarking algorithm is proved to be.

#### 5.4.1. Ablation Study

**Figure 11:**
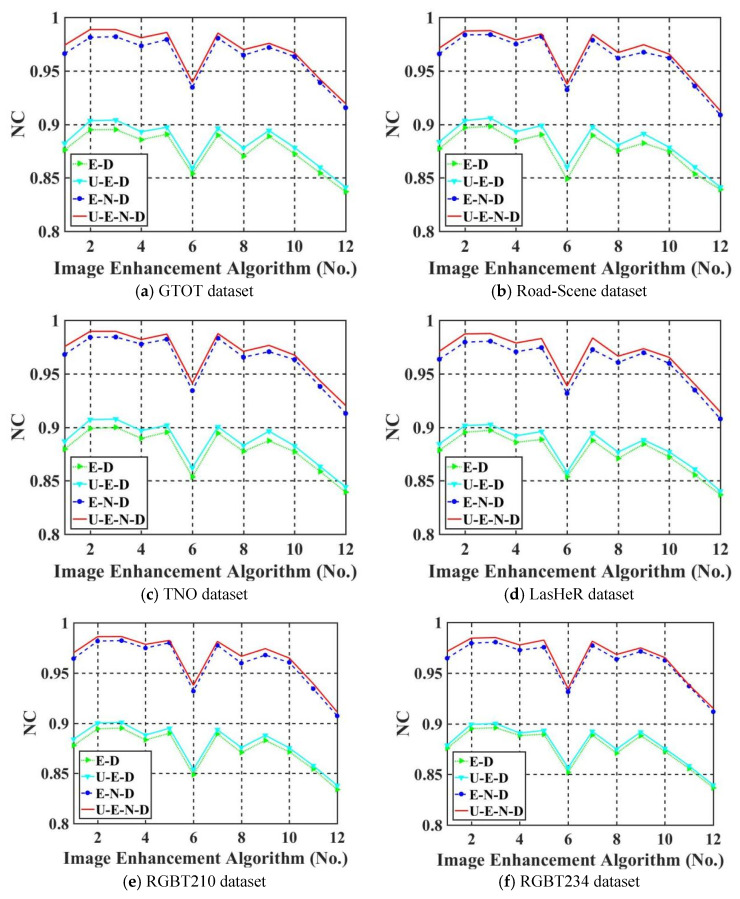
Comparison of average NC values for different model structures (**a**–**f**).

To verify the effectiveness of the proposed model components, we conduct a series of experiments according to the impact of UDTCWT [16] network U and noise network N on robustness. First, we remove UDTCWT [16] network U and noise network N from the proposed framework IEWNet, and then we test the robustness of the remaining structures against IRE attacks. Figure 11a–f shows Normalized Correlation (NC) values (as calculated in Equation (7)) of thewatermark extraction for different model structures on the six test datasets, respectively. The horizontal axis of the images represents the eight traditional-based IRE algorithms and four machine learning-based IRE algorithms in order.

(7) $$\mathrm{NC}=\frac{\mathrm{cov}\left(W_{in},W_{out}\right)}{\sigma \left(W_{in}\right)\times \sigma \left(W_{out}\right)},$$

where “cov” and “σ” denote the formulas for covariance and variance, respectively.

The structure U-E-N-D represents the complete structure of the proposed IEWNet model. E-D represents the encoder–decoder structure. E-N-D indicates the proposed IEWNet model after removing the UDTCWT [16] network U from its structure. U-E-D indicates the proposed IEWNet model after removing the noise network N. Figure 11 demonstrates that the noise network N improves a lot of the IRE attack’s robustness. The UDTCWT [16] network U also slightly enhances the NC value of watermark extraction (0 ≤ NC ≤ 1). The higher NC value is, the closer the image watermark $W_{\mathrm{out}}$ to $W_{\mathrm{in}}$ is.

#### 5.4.2. Comparative Study

A total of eight traditional-based IRE algorithms and four machine learning-based IRE algorithms are applied to test the robustness effect of the proposed IEWNet. The experiments use the same six datasets presented in Section 5.1. Furthermore, we compare our experimental results with four recent image robust watermarking algorithms: Cao et al. [46], Singh et al. [19], Anand [51], and Niu et al. [52], as shown in Table 1.

In order to verify the robustness of the algorithm from different perspectives, we use BER as a criterion in this section. The BER value indicates how many proportions of pixels of the embedded watermark $W_{\mathrm{in}}$ and extracted watermark $W_{\mathrm{in}}$ are different. Table 1 compares the average BER values for the six test sets. For the GTOT [40] dataset, the average BER value of IEWNet for all attacks is 1.50% and it outperforms the average BER values of the other comparing algorithms [18,46,51,52], which are: 2.25%, 1.81%, 2.53%, and 2.64%, respectively. The experimental results of the proposed model on Road-Scene [41] dataset show that the average BER value is about 1.62%. The average BER values of the algorithms used for comparison are 1.95%, 2.39%, 2.68%, and 2.83%, respectively. The robustness of IEWNet on the TNO [42] dataset (1.41%) is also better than Cao et al. [46] (1.73%), Singh et al. [20] (2.16%), Anand [51] (2.47%), and Niu et al. [52] (2.59%). For the LasHeR [43] dataset, the average BER value of IEWNet is 1.65% and it also outperforms the average BER values of the other four algorithms [18,46,51,52], which are: 2.41%, 1.99%, 2.71%, and 2.85%, respectively. The experimental results of the RGBT210 [44] dataset show that average BER value of our algorithm is about 1.70%. The average BER values of the algorithms used for comparison [18,46,51,52] are 2.45%, 1.99%, 2.74%, and 2.88%. The robustness of IEWNet on the RGBT234 [45] dataset (1.41%) is also better than Cao et al. [46] (1.99%), Singh et al. [19] (2.36%), Anand [51] (2.62%), and Niu et al. [52] (2.71%).

Table 2 shows the BER values of machine learning-based IRE attacks for the six IR image test sets. The experimental results demonstrate that the proposed IEWNet exhibits the lowest average BER value and the best robustness on all datasets.

### 5.5. Watermark Capacity Test

**Figure 12:**
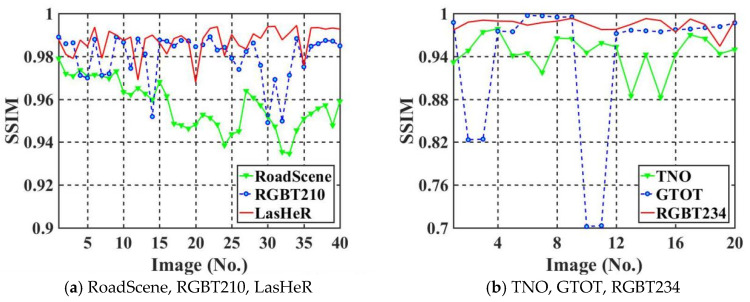
Comparison of SSIM values for different watermark capacities (**a**,**b**).

This section tests the watermark capacity of the proposed algorithm using six infrared image datasets. The watermark capacity is increased to 1024 bits. In order to demonstrate more fully the imperceptibility of the algorithm, we use Structural Similarity Index Measure (SSIM) to test the imperceptibility of the six IR images datasets. The computation of SSIM is shown in Equation (8).

(8) $$\mathrm{SSIM}=\frac{\left[2\times \mathrm{mean}\left(I_{em}\right)\times \mathrm{mean}\left(I\right)+c_{1}\right]\times \left[2\times \mathrm{cov}\left(I_{em},I\right)+c_{2}\right]}{\left[\mathrm{mean}{\left(I_{em}\right)}^{2}+\mathrm{mean}{\left(I\right)}^{2}+c_{1}\right]\times \left[\sigma {\left(I_{em}\right)}^{2}+\sigma {\left(I\right)}^{2}+c_{2}\right]},$$

where “mean” indicates the mean. And $c_{1}={\left(0.01\times 255\right)}^{2}$; $c_{2}={\left(0.03\times 255\right)}^{2}$. The SSIM values for each test set are shown in Figure 12.

After observing Figure 12 and all the IR images in the test set, we can find that the more smoothed the regions are in the IR images, the worse the imperceptibility of the watermarked IR images. Those IR images with very low PSNR or SSIM have much smoother regions than other IR images. Also, the brightness of the IR images does not affect PSNR and SSIM to the same degree. Thus, a too large difference in brightness between individual images and others can also lead to oscillations in the results.

With the watermark capacity of 1024 bits, Table 3 calculates the average BER values for the six IR datasets. The experimental results prove that the test sets GTOT and TNO with a small number of IR images have good robustness. The RGBT234 test set has some IR images with many texture features. This makes it slightly less robust than the previous two test sets. The robustness of the other three test sets is relatively close.

## 6. Discussion

IR images captured by existing IR image acquisition devices are difficult to use directly in industrial production and daily life. Therefore, IRE algorithms are often applied to process IR images. However, since IR image acquisition is expensive, IR images also need copyright protection. Previous research works usually use infrared images as the carrier to embed watermarking information without considering the effect of IRE attacks on watermarked images. The proposed algorithm is able to extract the watermark information after the watermarked IR image is enhanced. This is very helpful for industrial scenarios. In this paper, only IRE algorithm is targeted to improve the robustness of the algorithm. In future research work, more complex IR image processing algorithms should be considered. And more investigation should be done to improve the algorithm for the combined attack of multiple IR processing algorithms.

## 7. Conclusions

Due to the limitations of IR images, IRE algorithms are often used to improve the quality of IR images. However, these have a negative impact on the watermarking information embedded in IR images. In this paper, we propose an image robust watermarking auto-encoder network against IRE for both traditional-based and machine learning-based IRE attacks, namely IEWNet. The network employs UDTCWT [1] network to help the encoder and decoder to pre-extract the features from IR image. The multi-scale network structure used by the encoder and decoder is capable of extracting the features of the infrared image and the watermarking information at different scales. This is more conducive to discovering regions of the infrared image that are more suitable for embedding the watermark information. And the encoder also combines the original image and the watermarking information at the same scale, making the algorithm imperceptible. The network learns traditional and machine learning-based IRE algorithms to shape the noise layer and enhancement module, respectively. They can learn the laws of IRE attacks to help IEWNet extract image watermark information more accurately. However, the number and variety of IRE algorithms are very large, and they have different principles.

There is currently no proposed algorithm that can determine which IRE algorithm the enhanced IR image is sourced from, so an algorithm that can quickly and accurately analyze the type of IRE needs to be investigated in the future. And in future work, we should categorize the wide variety of IRE algorithms more precisely. Networks constructed based on these categories with different simulated IRE algorithms have the potential to achieve better imperceptibility and robustness. And there are other processing methods for infrared images in industrial scenarios, such as the special noise generated in the process of transmission. Developing novel and practical algorithms based on more complex industrial scenes in real life will be of great help to industrial production and scientific research.

## Figures and Tables

**Table 1:** Bit Error Ratio (BER) values of the traditional-based IRE attacks for six IR image datasets.

	(a) GTOT Dataset				
Attack	BER (%)				
	Niu et al. [52]	Anand [51]	Singh et al. [19]	Cao et al. [46]	Proposed
HE	2.6171	2.7343	2.5390	2.3632	1.6601
Mean Filter	1.7382	1.5625	1.0742	0.7812	0.7031
Median Filter	1.6601	1.5039	1.0546	0.7421	0.7031
Sobel Operator	1.9140	1.9531	1.8164	1.3085	1.2109
AHPBC	1.8554	1.7773	1.5429	1.1718	0.8789
DWT-EPSO	6.4257	6.2890	5.8007	4.7265	4.0625
DeepVIF	1.9726	1.7578	1.5625	1.2109	0.9179
ANFLC	2.9492	2.6953	2.6367	2.1875	1.9335
	(b) Road-Scene dataset				
Attack	BER (%)				
	Niu et al. [52]	Anand [51]	Singh et al. [19]	Cao et al. [46]	Proposed
HE	2.7734	2.8320	2.5683	2.5140	1.8359
Mean Filter	1.9140	1.6992	1.1816	0.8886	0.7910
Median Filter	1.8847	1.6503	1.1718	0.8789	0.7715
Sobel Operator	2.0996	1.9531	1.8945	1.3476	1.3281
AHPBC	2.0507	1.9238	1.7187	1.2792	0.9668
DWT-EPSO	6.7382	6.4843	5.8496	5.0195	4.1601
DeepVIF	2.1191	1.8847	1.8066	1.2988	0.9961
ANFLC	3.1347	3.0273	2.9394	2.4414	2.1191
	(c) TNO dataset				
Attack	BER (%)				
	Niu et al. [52]	Anand [51]	Singh et al. [19]	Cao et al. [46]	Proposed
HE	2.5585	2.5976	2.3828	2.3242	1.5625
Mean Filter	1.7187	1.5234	1.0156	0.7031	0.6445
Median Filter	1.6601	1.4843	0.9960	0.7031	0.6445
Sobel Operator	1.8750	1.8554	1.7578	1.1914	1.1328
AHPBC	1.8164	1.7187	1.4843	1.1328	0.8007
DWT-EPSO	6.3671	6.1914	5.6250	4.6484	3.9257
DeepVIF	1.9140	1.6796	1.5625	1.0937	0.7812
ANFLC	2.8515	2.7148	2.5195	2.0507	1.8554
	(d) LasHeR dataset				
Attack	BER (%)				
	Niu et al. [52]	Anand [51]	Singh et al. [19]	Cao et al. [46]	Proposed
HE	2.7929	2.8515	2.6074	2.5976	1.8652
Mean Filter	1.9335	1.6894	1.2304	0.8984	0.8007
Median Filter	1.9042	1.6601	1.2109	0.8984	0.7715
Sobel Operator	2.1093	1.9824	1.9042	1.3867	1.3476
AHPBC	2.0703	1.9335	1.7578	1.2792	1.0742
DWT-EPSO	6.7089	6.5234	5.8105	5.1074	4.1406
DeepVIF	2.1582	1.9628	1.8652	1.2890	1.1035
ANFLC	3.1738	3.1054	2.9004	2.4707	2.1582
	(e) RGBT210 dataset				
Attack	BER (%)				
	Niu et al. [52]	Anand [51]	Singh et al. [19]	Cao et al. [46]	Proposed
HE	2.8320	2.8613	2.6367	2.6074	1.9140
Mean Filter	1.9531	1.7187	1.2597	0.9082	0.8691
Median Filter	1.9043	1.6992	1.2207	0.8886	0.8593
Sobel Operator	2.1386	2.0019	1.9140	1.3574	1.3574
AHPBC	2.0800	1.9824	1.7285	1.2988	1.1035
DWT-EPSO	6.7871	6.5430	5.8691	5.0292	4.1992
DeepVIF	2.1875	2.0117	1.9531	1.4062	1.1719
ANFLC	3.2031	3.1152	3.0761	2.4902	2.1484
	(f) RGBT234 dataset				
Attack	BER (%)				
	Niu et al. [52]	Anand [51]	Singh et al. [19]	Cao et al. [46]	Proposed
HE	2.7148	2.8125	2.6757	2.4609	1.8554
Mean Filter	1.7773	1.6015	1.1718	1.0351	0.9765
Median Filter	1.7187	1.5625	1.1132	1.0156	0.9375
Sobel Operator	1.9921	2.0312	1.9335	1.5234	1.4062
AHPBC	1.8750	1.8164	1.6015	1.2695	1.0937
DWT-EPSO	6.5039	6.4062	5.9375	4.9218	4.4140
DeepVIF	2.0507	1.8164	1.6992	1.4062	1.1718
ANFLC	3.1250	2.9687	2.7929	2.3437	2.0312

**Table 2:** BER values for machine learning-based IRE attacks on the six IR image datasets.

	(a) GTOT Dataset				
Attack	BER (%)				
	Niu et al. [52]	Anand [51]	Singh et al. [19]	Cao et al. [46]	Proposed
TEN	3.2812	3.1250	2.4804	1.8554	1.5429
BCNN	3.8085	3.7695	2.7539	2.3046	2.1484
ONRDCNN	7.4609	7.0898	5.9765	4.9804	3.8671
ACNN	7.7929	7.5781	6.3671	6.1718	5.5859
	(b) Road-Scene dataset				
Attack	BER (%)				
	Niu et al. [52]	Anand [51]	Singh et al. [19]	Cao et al. [46]	Proposed
TEN	3.3789	3.2324	2.5488	2.0117	1.6211
BCNN	4.1210	4.0722	3.0175	2.4707	2.2168
ONRDCNN	7.6171	7.4902	6.3574	5.3515	4.0136
ACNN	8.5058	8.2128	6.8847	6.5527	5.9667
	(c) TNO dataset				
Attack	BER (%)				
	Niu et al. [52]	Anand [51]	Singh et al. [19]	Cao et al. [46]	Proposed
TEN	3.1640	3.0468	2.3046	1.7382	1.4843
BCNN	3.7304	3.5546	2.7148	2.2460	2.0898
ONRDCNN	7.3828	7.0117	5.8203	4.9218	3.7695
ACNN	7.6367	7.4804	6.2890	6.1328	5.4687
	(d) LasHeR dataset				
Attack	BER (%)				
	Niu et al. [52]	Anand [51]	Singh et al. [19]	Cao et al. [46]	Proposed
TEN	3.4375	3.2714	2.5683	2.0312	1.6992
BCNN	4.2188	4.2089	3.0273	2.4218	2.2363
ONRDCNN	7.6660	7.5488	6.3964	5.3710	4.0234
ACNN	8.7402	8.2910	6.9140	6.6992	6.0059
	(e) RGBT210 dataset				
Attack	BER (%)				
	Niu et al. [52]	Anand [51]	Singh et al. [19]	Cao et al. [46]	Proposed
TEN	3.5449	3.5058	2.6269	2.0605	1.6406
BCNN	4.2578	4.2383	3.0273	2.5097	2.2656
ONRDCNN	7.8417	7.6563	6.4746	5.4395	4.0820
ACNN	8.9063	8.6621	6.9727	6.8945	6.0937
	(f) RGBT234 dataset				
Attack	BER (%)				
	Niu et al. [52]	Anand [51]	Singh et al. [19]	Cao et al. [46]	Proposed
TEN	3.6328	3.4375	2.7343	2.0507	1.6015
BCNN	3.9453	4.0429	2.8125	2.4414	2.2460
ONRDCNN	7.5976	7.1875	6.1523	5.1757	4.1601
ACNN	7.9492	7.8125	6.5039	6.3085	5.9179

**Table 3:** Average BER value (%) for 1024-bit watermark capacity.

(a) Proposed						
Attack	GTOT	Road-Scene	TNO	LasHeR	RGBT210	RGBT234
HE	3.9746	4.3383	4.0673	6.5429	5.9521	7.4560
Mean Filter	1.9677	2.2363	1.8554	3.5986	3.4350	3.8769
Median Filter	1.7431	2.0263	1.7675	3.2812	3.0078	3.6279
Sobel Operator	3.6425	3.2275	3.0371	5.1416	4.4653	5.7421
AHPBC	2.0166	2.2949	2.1240	4.5507	3.7304	4.4677
DWT-EPSO	9.1650	10.4370	8.9648	13.3447	12.3046	13.8769
DeepVIF	2.3730	2.4291	2.3437	4.8754	3.9111	5.0830
ANFLC	4.9609	5.1513	5.6396	8.7329	7.6708	9.1308
TEN	4.1162	3.9868	3.4423	5.5664	5.0122	6.0302
BCNN	4.5605	5.1293	4.4238	8.2104	7.0922	7.9589
ONRDCNN	8.3789	10.1391	8.7060	12.4682	11.8334	13.1689
ACNN	13.1152	14.1577	12.5732	18.0249	17.2924	18.9550
(b) Cao et al. [46]						
Attack	GTOT	Road-Scene	TNO	LasHeR	RGBT210	RGBT234
HE	7.7881	8.3105	7.1533	8.7305	9.0430	10.3418
Mean Filter	2.6074	3.1982	2.1826	3.5742	3.5547	4.0967
Median Filter	2.3438	3.0615	2.1387	3.3447	3.2178	3.6719
Sobel Operator	4.9756	5.3223	4.1406	6.0986	5.3223	6.8115
AHPBC	3.8672	4.9121	3.3789	5.3711	4.3994	4.6045
DWT-EPSO	12.4951	15.8545	12.0947	17.0898	16.4453	17.5684
DeepVIF	3.5498	4.4824	3.1689	4.1504	5.5273	6.4893
ANFLC	7.1104	8.9756	6.1094	9.2588	9.6494	10.1172
TEN	4.8438	6.0156	4.4678	6.7676	7.6074	7.2705
BCNN	5.9570	9.5068	5.5762	8.9404	9.9121	9.6191
ONRDCNN	11.5918	14.1064	11.0791	14.7363	15.4785	15.6934
ACNN	14.8730	20.1660	14.5117	20.6299	21.0352	20.6582
(c) Singh et al. [19]						
Attack	GTOT	Road-Scene	TNO	LasHeR	RGBT210	RGBT234
HE	8.2617	8.9697	7.6221	9.3896	9.8926	11.7188
Mean Filter	2.9053	3.3643	2.2852	3.9307	4.1406	4.5264
Median Filter	2.8711	3.2373	2.1045	3.4229	3.7939	4.0967
Sobel Operator	5.7031	5.4492	4.9365	7.2266	7.6758	8.5791
AHPBC	5.3320	5.8691	4.6289	6.9287	6.3623	7.4707
DWT-EPSO	14.5654	15.7275	13.6816	17.1680	16.0156	17.4365
DeepVIF	4.6924	5.7715	3.8428	6.2012	6.9043	8.0859
ANFLC	8.7842	9.5850	8.1445	9.1650	10.1807	9.8145
TEN	5.9326	6.8945	5.0439	7.6367	8.3301	9.0869
BCNN	7.6465	9.1455	6.1963	9.9756	9.6289	10.5811
ONRDCNN	12.8418	14.2139	11.9092	16.1621	16.6406	17.2705
ACNN	16.7725	18.4180	15.6787	20.9619	21.6162	22.3291
(d) Anand [51]						
Attack	GTOT	Road-Scene	TNO	LasHeR	RGBT210	RGBT234
HE	12.6709	13.9355	11.7627	14.3652	15.2979	16.1328
Mean Filter	5.1465	6.8652	4.8291	6.3965	7.9541	8.4277
Median Filter	4.4092	5.9229	3.7598	6.0547	6.7139	7.5879
Sobel Operator	9.4824	9.7852	8.5742	10.5859	11.2061	11.9336
AHPBC	7.5781	8.1348	7.2852	8.8916	9.3262	9.9268
DWT-EPSO	19.4482	21.8506	18.3740	22.6855	23.6523	25.0928
DeepVIF	7.9639	8.7012	7.2559	9.5361	10.0928	10.4150
ANFLC	11.6260	12.9199	12.2412	13.8770	14.2041	15.6934
TEN	9.7803	11.6260	8.7549	11.9385	13.3105	14.3408
BCNN	14.5898	15.3809	13.6670	16.1621	16.7432	17.7295
ONRDCNN	18.0664	20.1807	17.4023	21.0107	21.9629	23.3105
ACNN	21.3574	24.4092	19.7949	25.2051	26.7627	29.0918

## Data Availability

Dataset available on request from the authors. The datasets used in this paper include GTOT, Road-Scene, TNO, LasHeR, RGBT210 and RGBT234. These datasets are available for download with links in their references or on the official website of the author’s team. https://github.com/mmic-lcl/Datasets-and-benchmark-code. The datasets are allowed to be used in non-commercial activities.

## Abbreviations

The following abbreviations are used in this manuscript:

IR	Infrared
IRE	Infrared Enhancement
UDTCWT	Undecimated Dual Tree Complex Wavelet Transform
PSNR	Peak Signal to Noise Ratio
HE	Histogram Equalization
CNN	Convolutional Neural Networks
ReLU	Rectified Linear Unit
GAN	Generative Adversarial Network
FFT	Fast Fourier Transform
DWT	Discrete Wavelet Transform
DNN	Deep Neural Network
DNST	Discrete Non-separable Shearlet Transform
PZM	Pseudo-Zernike Moments
DCT	Discrete Cosine Transform
WHT	Walsh Hadamard Transform
SVD	Singular Value Decomposition
GBT	Graph Based Transform
NCWT	Nondownsampled Contour Wave Transform
TEN	Thermal Image Enhancement using CNN
BCNN	Brightness-Based CNN
ONRDCNN	Optical Noise Removal Deep CNN
ACNN	Auto-Driving CNN
AHPBC	Adaptive Histogram Partition and Brightness Correction
DWT-EPSO	Discrete Wavelet Transform and Event triggered Particle Swarm Optimization
ANFLC	Adaptive Non-local Filter and Local Contrast
SGD	Stochastic Gradient Descent
HF	high-frequency
LF	low-frequency
NC	Normalized Correlation
SSIM	Structural Similarity Index Measure
